# Nance–Horan syndrome pedigree due to a novel microdeletion and skewed X chromosome inactivation

**DOI:** 10.1002/mgg3.2100

**Published:** 2022-11-12

**Authors:** Yazhou Huang, Linya Ma, Zhaoxia Zhang, Shujuan Nie, Yuan Zhou, Jibo Zhang, Chao Wang, Xingxin Fang, Yingting Quan, Ting He, Anhui Liu, Dan Peng

**Affiliations:** ^1^ Department of Medical Genetics Changde First People's Hospital Changde China; ^2^ Affiliated Hospital of Changde City University of South China Hengyang China

**Keywords:** copy number variation sequencing, dense congenital cataracts, genetic counseling, Nance–Horan syndrome, X chromosome inactivation

## Abstract

**Background:**

Nance–Horan syndrome (NHS) is a rare and often overlooked X‐linked dominant disorder characterized by dense congenital cataracts, dental abnormalities, and mental retardation. The majority of NHS variations include frameshift mutations, nonsense mutations, microdeletions, and insertions.

**Methods:**

Copy number variation sequencing was performed to determine the microdeletion. The expression of *NHS* was detected by RT‐PCR. Four family members were tested for X chromosome inactivation.

**Results:**

In this study, all members were examined for systemic examinations and genetic testing of four members and two affected subjects are observed. We identified a heterozygous microdeletion of −0.52 Mb at Xp22.13 in a female proband presenting NHS phenotypically. The microdeletion contains the *REPS2* and *NHS* genes and was inherited from a phenotypically normal mother. Of interest, the expression *NHS* of proband was reduced and the skewed X chromosome inactivation rate reached more than 85% compared with her mother and the control. It was concluded that the haploinsufficiency of the *NHS* gene may account for the majority of clinical symptoms in the affected subjects. The variability among female carriers presumably results from nonrandom X chromosome inactivation.

**Conclusion:**

Our findings broaden the spectrum of *NHS* mutations and provide molecular insight into NHS clinical prenatal genetic diagnosis.

## Graphical abstract

Firstly, we identified a heterozygous microdeletion of 0.52 Mb at Xp22.13 detected by genome‐wide copy number variation sequencing in a proband presenting congenital cataract, facial dysmorphisms, and mental retardation. Then, the pathogenesis was explored from gene expression level and X chromosome inactivation patterns. Lastly, it was concluded that the haploinsufficiency of the NHS gene may account for the majority of clinical symptoms in the affected subjects.

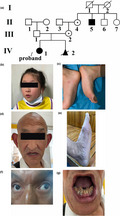

## 1 INTRODUCTION

The Nance–Horan syndrome (NHS) (OMIM 302350) is a rare X‐linked developmental disorder characterized by bilateral severe congenital cataracts, dental anomalies such as screwdriver‐shaped teeth and bud molars, dysmorphic facial features such as long and narrow face, anteverted pinnae, and broad nose (Horan & Billson, 1974; Nance et al., 1974). Varying levels of intellectual disability and behavioral disturbance may be seen in approximately 30% of male patients (Burdon et al., 2003; Sharma et al., 2017). Affected patients are typically males while carrier females are mild or asymptomatic (Goodman et al., 1965). Fewer than 50 NHS families have been described in the scientific literatures according to National Organization for Rare Disorders. (https://rarediseases.org/rare‐diseases/nance‐horan‐syndrome/).

NHS is caused by mutations in the *NHS* gene on chromosome Xp22 (Burdon et al., 2003), which comprises nine coding exons and encodes at least seven isoforms as a result of alternative splicing (Brooks et al., 2010). The *NHS* gene encompasses ~650 kb of genomic DNA, mainly coding for NHS‐A (NM_198270) and NHS‐1A (NM_001291867) protein. The two isoforms are transcribed from exon 1 of the *NHS* gene, encoding a 1630‐amino acid protein which may interact with the tight junction protein zona occludens‐1 and a 1651‐amino acid protein, respectively (Brooks et al., 2010; Sharma et al., 2009). The NHS nuclear protein acts as a regulator of actin remodeling and cell morphology, which is essential for mediating changes in cell shape, migration, and cell contact (Brooks et al., 2010). Meanwhile, they might have a crucial function in the regulation of brain, lens, dental primordia, and craniofacial development (Brooks et al., 2004, 2010; Burdon et al., 2003). To date, it is interesting that the most pathogenic mutations associated with *NHS* are frameshift and nonsense mutations that result in either nonsense‐mediated decay of the mRNA or truncation of the NHS protein (Florijn et al., 2006; Huang et al., 2007; Reches et al., 2007; Sharma et al., 2008). In addition, a few microdeletions and insertions have been reported, some of which encompass also other genes, such as the *REPS2*, *SCML1*, and *RAI2* genes (Liao et al., 2011).

X chromosome plays a significant role in many concomitant genetic diseases. X‐chromosome inactivation (XCI) of females for dosage compensation of X‐linked genes between sexes, by which either the paternal or maternal X is randomly chosen for silencing in each cell. XCI is initiated during early female mammalian embryonic development (Payer & Lee, 2008), by allele‐specific upregulation of the long noncoding RNA Xist from the future inactive. Once established, the XCI can be stably inherited in the differentiated cells (Okamoto et al., 2004). Therefore, any significant deviation from this mosaic state is termed as skewed X‐chromosome inactivation (SXCI), which is closely related to clinical disease and is a hot topic of medical research (Brockdorff et al., 1991; Brown et al., 1991; Penny et al., 1996). It is usually the structurally abnormal X chromosome that is preferentially inactivated (Baruffi et al., 2012; Ferrier et al., 1965; Fonova et al., 2022), but, normal X chromosome inherited from her father is preferentially inactivated which resulted in down‐regulation of *NHS* gene expression in the event of disease as mentioned in this article.

In this study, we describe the clinical and genetic analysis of a heterozygous microdeletion of −0.52 Mb at Xp22.13 detected through high‐throughput genome‐wide copy number variation sequencing(CNV‐seq). Clinical NHS pedigree was found and the same fragments of *NHS* gene were deleted in the family. However, there were significant differences in clinical diagnosis phenotypes. The proband showed severe congenital cataract, characteristic dysmorphic features, dental anomalies, and mental retardation, while the carrier mother and grandmother had normal intelligence, no cataract symptoms, and completely normal eyes. We demonstrate that NHS disease development, clinical phenotypes, or outcomes is associated with SXCI. Furthermore, we conclude that *NHS* heterozygous microdeletion along with preferential X‐inactivation causes familial NHS, and summarize the relationship between *NHS* major isoforms expression and disease phenotype expressivity.

## 2 MATERIALS AND METHODS

### 2.1 Editorial policies and ethics statement

A written informed consent was obtained from each subject or their guardians to participate in this study. The study was conducted according to the guidelines of the Declaration of Helsinki, and approved by the Ethics Committee of Changde First People's Hospital (protocol code 2021‐220‐01) and approved on September 28, 2021.

### 2.2 Pedigree

**FIGURE 1:**
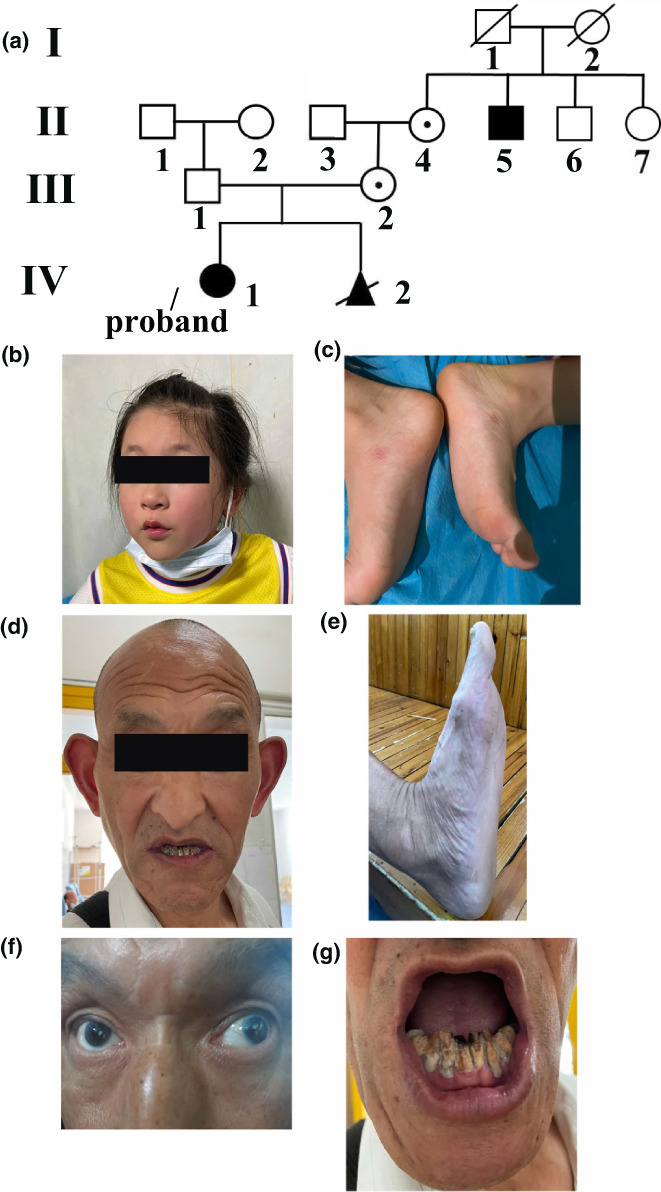
Pedigree structure and patient phenotypes of the family. (a) The black solid square and circle represents the affected male patient and female patient, respectively. The black circles with dots in the middle represents the female carrier. Unfilled squares and circles indicate normal males and females, respectively. (b) Proband (IV‐1) and her granduncle (II‐5) (d) representative facial dysmorphology of the NHS patients. IV‐1 and II‐5 had a long and narrow face, anteverted, and mild enlarged pinnae and bulbous nose, respectively. (c) IV‐1 and II‐5 (e) had flat foot abnormalities

The NHS family was ascertained through the medical genetics clinic of Changde First People's Hospital. There are five males, five females, and an unborn boy in this family who participated in the study. The parents were unrelated, healthy, and did not receive medical attention until they delivered the proband (Figure 1a IV‐1) who is a 5‐year‐old girl affected with congenital cataract and developmental abnormalities. Otherwise, proband's granduncle had a long‐narrow face, prominent nose, and large anteverted pinnae, screw‐driver like incisors. Other members of the NHS family were asymptomatic and associated with the NHS.

### 2.3 Karyotyping and copy number variation sequencing

The peripheral blood cells were harvested and prepared after 3 days of culture in the CO_2_ incubator. 180 fission images were observed and counted under the microscope after G banding by using ZEISS Metasystems (Germany), and five karyotypes were analyzed.

Genomic DNA was isolated from peripheral blood cells using the DNeasy Blood & Tissue Kit (Qiagen) according to the manufacturer's instructions. CNV‐Seq was performed using 50 ng of genomic DNA as the starting template. In brief, for the construction of sequencing libraries, DNA was first fragmented to an average size of 300 bp, ligated with 9 bp barcoded sequencing adaptors, and the modified fragments amplified by PCR. After PCR amplification, the fragment selection and purification were carried out by magnetic bead purification method to remove the interference of primer dimer in the reaction system, so as to obtain the DNA library. CNV‐seq was performed Illumina HiSeq2500 platform (Illumina, Inc., San Diego, CA, USA). Copy number gains or losses were compared without in‐house database of copy number variants (CNVs) and with public CNV databases, including Genomic Variants (http://dgv.tcag.ca/dgv/app/home), Decipher (http://decipher.sanger.ac.uk/), and ClinGen (https://www.clinicalgenome.org/). All genomic coordinates are based on the Human GRCh37/hg19 Genome Assembly. The American College of Medical Genetics and Genomics (ACMG 2019) standard was used as the final criterion to evaluate the pathogenicity of the microdeletion. If abnormal CNV changes of unknown clinical significance were detected in the peripheral blood sample, the parental samples were analyzed for the aberration.

### 2.4 Quantitative real‐time PCR and X chromosome inactivation analysis

Total RNA was isolated using QIAamp RNA Blood Mini Hand (QIAamp), and reverse transcription reactions were performed using PrimeScript RT reagent kit with gDNA Eraser (TaKaRa), according to the manufacturer's protocol. Primers (unique shared region of *NHS*‐A and *NHS*‐1A forward primer 5′‐GAGCTCGAGAGCGACATCCA‐3′; reverse primer 5′‐CCTGCTTAGGGTCAAGCGT‐3′, homologous segments of seven *NHS* transcript isoforms forward primer 5′‐ATTGTGCACACAAACCCAGA‐3′; reverse primer 5′‐CAGAAATGTTGCCAGCAGAA‐3′) were designed using primer 3 software. *GAPDH* (forward primer 5′‐GTCAAGGCTGAGAACGGGAA‐3′, reverse primer 5′‐TCGCCCCACTTGATTTTGGA‐3′) was used as housekeeping gene. The amplification was performed under the following conditions: 95°C for 5 min, followed by 40 cycles at 95°C for 30 s, 58°C for 30 s, 72°C for 30 s. qRT‐PCR was performed in triplicates of each condition, using SyBR green (Roche) and a LineGene 9600 PCR system (LineGene). The relative expression level of *NHS* gene was calculated according to 2^−ΔΔCT^.

Genomic DNA was extracted using whole blood DNA extraction kit (Tiangen Biotechnology, Beijing, China) and is predigested with a methylation‐sensitive restriction endonuclease, *Hpa*II. *Hpa*II will only cleave the DNA recognition site when the adjacent CpG island is undermethylated, hence, only the active human androgen receptor gene on the active X chromosome is digested. Following the DNA restriction digestion, the predigested samples are amplified by PCR, and only regions that are methylated, therefore, undigested, will amplify successfully. PCR products from the *Hpa*II‐digested genomic DNA are then compared to a separate aliquot of the same DNA amplified without *Hpa*II digestion. Androgen receptor alleles primers (forward primer 5′‐GCTGTGAAGGTTGCTGTTCCTCAT‐3′, reverse primer 5′‐TCCAGAATCTGTTCCAGAGCGTGC‐3′) are from Jones's articles (Jones, 2014). Comparison of peak areas of the two androgen receptor alleles provides data sufficient to determine X inactivation patterns in the original cell population from which the genomic DNA was derived. Samples that show one androgen receptor allele preferentially surviving *Hpa*II digestion are then determined to have nonrandom or “skewed” X inactivation. All samples were analyzed in duplicate, and the average of the ratios was taken. The XCI patterns were classified as random inactivation (50:50 to 80:20), moderately skewed inactivation (80:20 to 90:10), or highly skewed inactivation (90:10 and greater) (Eggan et al., 2000). Allele 1 inactivation rate = (Bd1/Bu1)/(Bd1/Bu1 + Bd2/Bu2, Bd1, Bd2): area of allele scanning peak after digestion of *Hpa*II endonuclease, Bu1, Bu2: the area of allele scanning peak without *Hpa*II endonuclease digestion.

### 2.5 *NHS* exon1 sanger sequencing

Sanger sequencing using an ABI PRISM 3730 DNA Sequencer (Applied Biosystems, Tsingke Biotechnology) was performed to validate the *NHS* exon1 variant in the proband as well as her parents using designed primers (forward primer 5’‐CACCACGCTGACAGTCTCAA‐3′, reverse primer 5’‐GGTGGGAAGGCGAGAGTAGT‐3′). Sequence analysis was performed by comparing sequence data with the reference genome using chromas and DNASTAR software.

## 3 RESULTS

### 3.1 Clinical findings

A 5‐year‐old girl was admitted to the Department of Medical Genetics of Changde First People's Hospital for genetic counseling due to congenital cataracts and her mother for genetic counseling of prenatal diagnosis. The pedigree of the family is shown in Figure 1a. Available clinical information regarding the families is shown in Table 1. Two affected subjects (Figure 1b:II‐5, d:IV‐1) available at the time of clinical examination presented with dental abnormalities, mental retardation, and bilateral congenital cataracts, one of whom received cataract surgery at the age of 4 years. Typical ocular features of NHS recorded in the affected subjects include microcornea, nystagmus, high myopia, and strabismus (Figure 1f). In addition, proband's granduncle presented with screwdriver‐shaped incisors (Figure 1g). Characteristic facial features of a long narrow face and prominent nose were noted in both affected subjects, and flat foot was exhibited (Figure 1c,e). However, heterozygous carrier females of family were asymptomatic (Figure 1a:II‐4, III‐2).

**TABLE 1:** Clinical features of affected and carrier members of the NHS family

Disease phenotype	Affected patients		Female carriers	
	IV‐1	II‐5	III‐2	II‐4
Ocular features				
Bilateral congenital cataract	+	+	−	−
Nystagmus	+	+	−	−
High myopia	+	+	−	−
Strabismus	+	+	−	−
Microcornea	+	+	−	−
Facial abnormalities				
Long‐narrow face	+	+	−	−
Anteverted and mild enlarged pinnae	+	+	−	−
Bulbous nose	+	+	−	−
Dental abnormalities				
Mulberry‐like molars	+	+	−	−
Screw‐driver shaped incisors	+	+	−	−
Hand and foot abnormalities	+	+	−	−
Mental retardation	+	+	−	−

### 3.2 Copy number variation sequencing identified an NHS microdeletion

**FIGURE 2:**
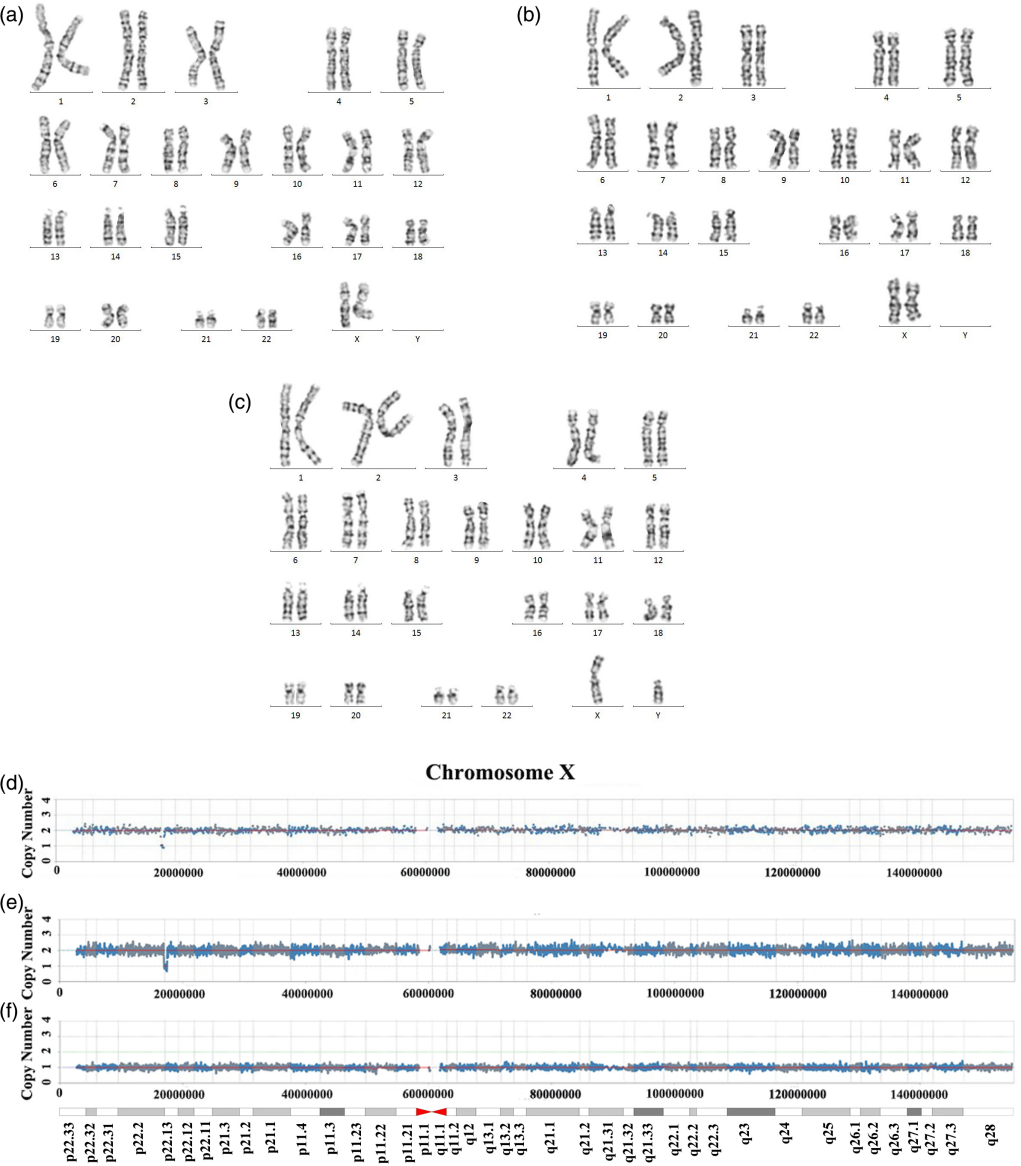
Chromosome G‐banding analysis and copy number variation analysis. IV‐1 (a), III‐2 (b), and III‐1 (c) chromosome G‐banding analysis was normal. IV‐1 (d) and III‐2 (e) showed a − 0.52 Mb microdeletion at chromosome Xp22.13, while III‐1 (f) was no deletions

**FIGURE 3:**
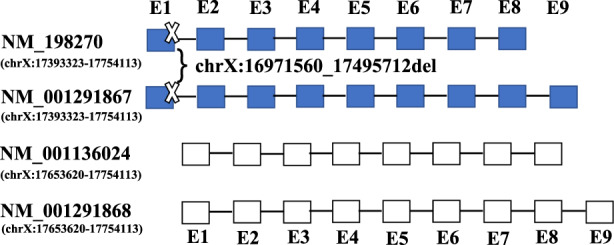
Schematic diagram of *NHS* gene isoforms by searching the bioinformatics website. The blue solid squares and unfilled squares represent the exon. The black line represents intron. The identified variation in this study causes the microdeletion (chrX:16971560_17495712del) of exon 1 and part of intron 1 of the *NHS* gene isoform 1 (NM_198270) and isoform 3 (NM_001291867), while having no effect on the other *NHS* gene isoforms

For this study, the proband, her mother (III‐1) and her father (III‐2) karyotype analysis was normal (Figure 2a–c). Copy number variation sequencing showed a microdeletion at chromosome Xp in proband (IV‐1). It was found that the proband inherited a deletion from her mother at nucleotide positions chrX:16996657–17394455, with a length of −0.52 Mb, including the *REPS2* and *NHS* genes at the cytogenetic band Xp22.13 (Figure 2d,e) (GRCH37/hg19). However, copy number variation analysis of her father was normal. The identified variation causes the deletion of exon 1 and part of intron 1 of the *NHS* gene isoform NM_198270 and isoform NM_001291867, while having no effect on the other gene isoforms by searching the bioinformatics website (https://genome.ucsc.edu) (Figure 3). Otherwise, it was found that there was no mutation or deletion in exon 1 of another normal chromosome through Sanger sequencing (data not shown). Therefore, it was concluded that the haploinsufficiency of the *NHS* gene may account for the majority of clinical symptoms in the affected subjects (https://dosage.clinicalgenome.org/).

### 3.3 *NHS* gene expression and X chromosome inactivation analysis

**FIGURE 4:**
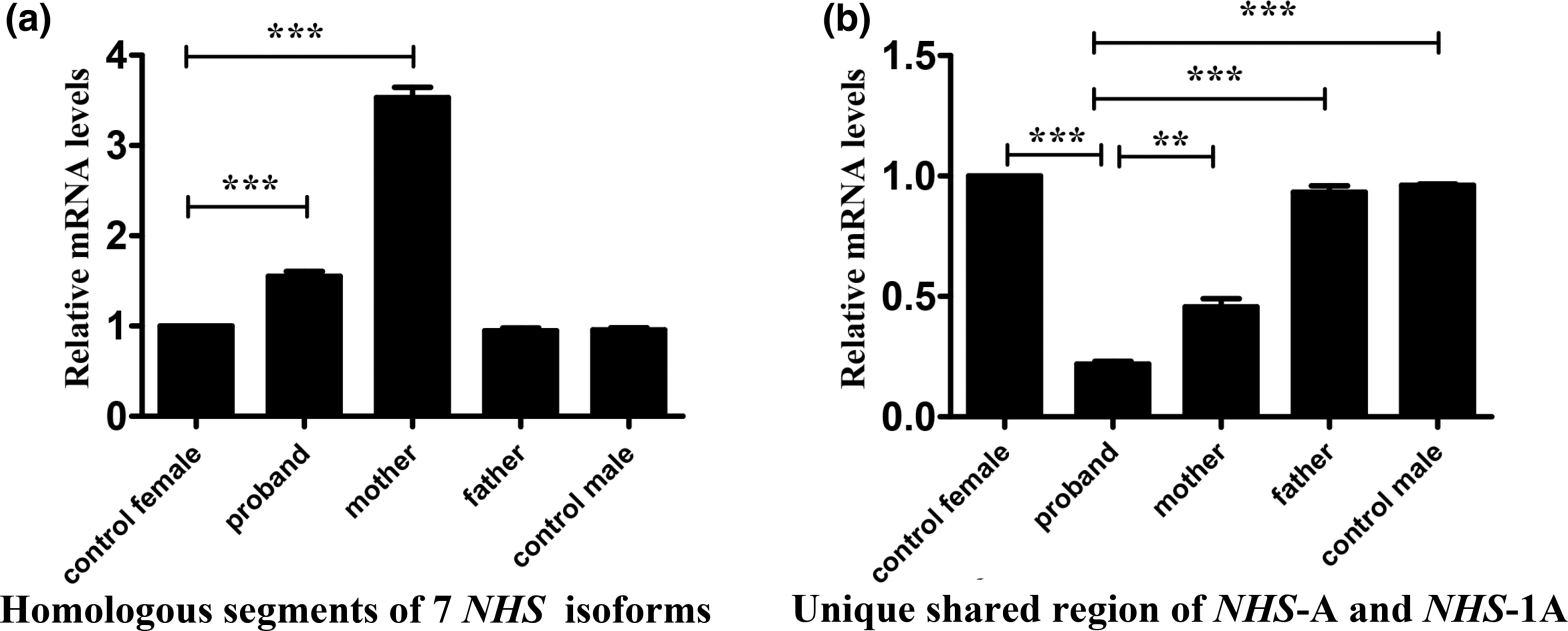
Real‐time quantitative PCR analysis of the *NHS* gene expression of the peripheral blood cells compared with the control. *p < 0.05, **p < 0.01, ***p < 0.001

**FIGURE 5:**
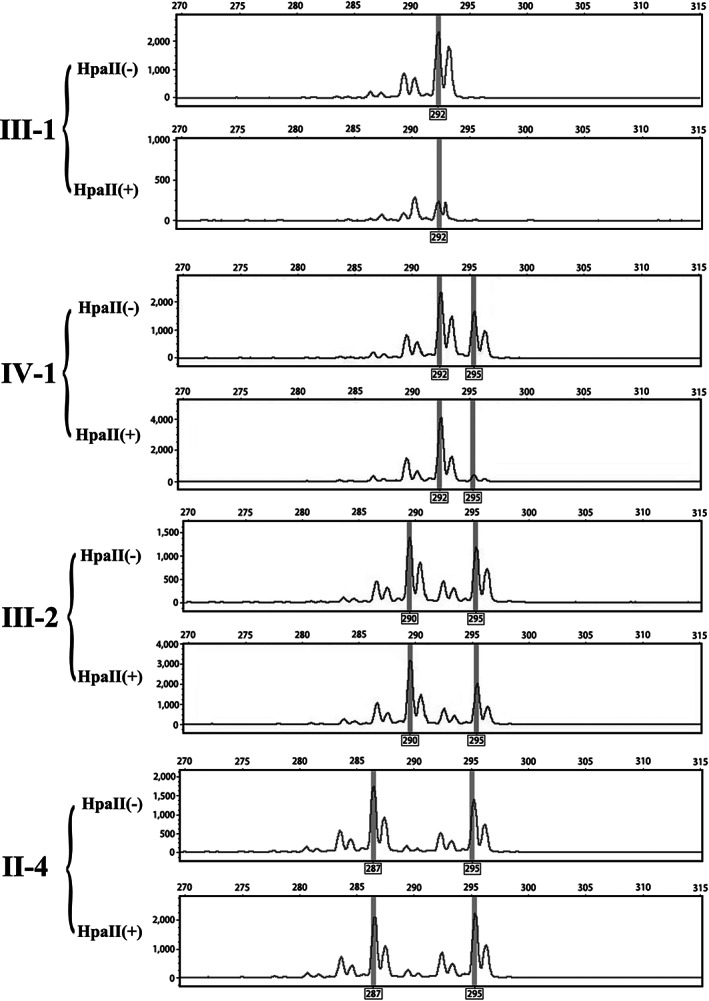
XCI pattern analysis using gDNA extracted from peripheral blood. Peaks indicate the amplified androgen receptor allele. The size of the allele is determined by the number of repeats in the exon1 of AR. *Hpa*II(−) and *Hpa*II(+) represent gDNA that was not digested with HpaII restriction endonuclease, and gDNA was digested with *Hpa*II restriction endonuclease, respectively. The father of proband indicates the high digestion efficiency of *Hpa*II restriction endonuclease. After the gDNA digested with *Hpa*II, the paternal allele (292 bp) of the proband (IV‐1) was almost entirely absent, which indicated that the female patient exhibited moderately skewed patterns of XCI. III‐2 and II‐4 XCI patterns were classified as random inactivation (Table 2)

To assess whether microdeletion of the *NHS* gene affects mRNA transcription, we performed an RT‐PCR experiment using RNA extracted from peripheral blood cells for the pedigree and the control. *NHS* gene transcripts isoform A and 1A of proband obviously reduced compared to control male or female (Figure 4b). When primers were designed for homologous segments of seven transcripts of *NHS* gene, the total *NHS* gene expression levels of proband and her mother were statistically different from those of the control (Figure 4a). Meanwhile, X‐inactivation analysis using peak areas assay showed that the normal chromosome X from father is preferentially inactivated in the proband. Compare to her mother (II‐2) and grandmother (II‐4), the skewed X chromosome inactivation rate of proband reached more than 85% (Figure 5 and Table 2). Skewed X‐chromosome inactivation of proband amplified haploinsufficiency effect of the *NHS* gene and lead to the symptoms of the disease while her mother and grandmother were asymptomatic. Finally, we speculate that there is an RNA compensation mechanism that results in higher gene expression than controls in mother and grandmother, but RNA compensation of proband was not enough to compensate for the skewed X chromosome inactivation effect.

**TABLE 2:** X‐chromosome inactivation patterns of peripheral blood

Family member	XCI pattern ratios (%)
IV‐1	88.5: 11.5
III‐2	58.7: 41.3
II‐4	45.5: 54.5

## 4 DISCUSSION

This study describes the clinical and molecular genetic analysis of a Chinese family exhibiting NHS. To date, in addition to most pathogenic frameshift or nonsense mutations that result in truncation of the NHS protein, small deletion and insertion mutations involving *NHS* gene at the coding sequences have been reported (Moysés‐Oliveira et al., 2015). A microdeletion of 2.8 Mb at Xp22 involving the *NHS* gene in a boy with severe encephalopathy, tetralogy of Fallot and bilateral congenital cataracts were firstly reported in 2007 (Van Esch et al., 2007). Subsequently, a homozygous mutation with a microdeletion of 0.92 Mb in *NHS* has been previously reported in a family with two affected Taiwanese brothers and their carrier mother (Liao et al., 2011). Coccia reported a deletion of 0.9 Mb at Xp22 comprising *NHS*, *RAI2, SCML1*, and *CXorf20* genes in a family with typical features of NHS syndrome and intellectual disability (Coccia et al., 2009). In summary, previous studies illustrated that microdeletion in the *NHS* gene could induce NHS. However, the identical association between the genotype and phenotype was not quite clear. Our study suggested that skewed X‐chromosome inactivation and aberrant transcription were possible causes of significantly phenotypic heterogeneity of NHS in carrier females. Otherwise, prenatal diagnosis of the fetus (Figure 1a IV‐2) was carried out and it was found to be a case of NHS.

X chromosome inactivation (XCI) is a key developmental process taking place in female mammals to compensate for the imbalance in the dosage of X‐chromosomal genes between sexes (Patrat et al., 2020). The XCI is a random process which the two X chromosomes from mother and father have an equal chance to be inactivated (Wu & Sun, 2016). Any significant deviation from this mosaic state is termed as skewed X‐chromosome inactivation (SXCI), which is clinically relevant. Miller describes the clinical implication of *NHS* gene disruption due to balanced X‐autosome translocations as a unique mechanism causing NHS, which refines dose effects of *NHS* on disease presentation and phenotype expressivity (Miller et al., 2021). Previous case reports suggest that NHS carrier females manifest mild and variable phenotypes. In individuals with abnormity involving the X chromosome, the abnormal X chromosome is preferentially inactivated. However, a preferential inactivation of the normal X chromosome has been reported to cause X‐linked disorders in the female carriers (Watanabe et al., 2018). Meanwhile, our study provides additional evidence of proband with preferential inactivation of normal chromosome X inherited from her father resulting in NHS phenotype while her carrier mother is asymptomatic.

The pathogenesis of genetic diseases is the primary issue to be considered in medical research. NHS is a syndrome with high phenotypic heterogeneity. Previous studies have shown that the phenotypes of patients with different mutations and found no obvious genotype–phenotype correlations between the position of each mutation and clinical severity (Tug et al., 2013). Traditionally, Whole‐exome sequencing followed by Sanger sequencing of variants identified mutation site in the *NHS* gene (Hong et al., 2014). In this study, we identified copy number variation of the *NHS* gene in the family using copy number variations sequencing. A segmental deletion encompassing the *NHS* and *RESP2* genes (0.52 Mb) was identified in the family, it is therefore likely that this region harbors important promoter sequence elements required for the spatial and temporal expression of NHS during development, and that disruption of the promoter leads to aberrant transcription of one copy of the *NHS* gene in the affected members of this family. The copy of the *NHS* gene lacks exon 1, therefore, isoform A and isoform 1A will not be expressed. Although the female‐affected individuals had the same variation, the phenotypic heterogeneity existed in the NHS family. We suggested that a lack of *NHS* mRNA expression might cause NHS, whereas aberrant transcription of the *NHS* gene might lead to a milder phenotype (https://cat‐map.wustl.edu/). Proband has NHS isoform A and 1A expression lower than the control but not completely absent. In summary, we predicted that skewed X‐inactivation and aberrant transcription of the *NHS* give rise to phenotypic heterogeneity in the family.

## 5 CONCLUSIONS

In this study, we found a novel microdeletion variation in the *NHS* gene, which was predicted to be likely associated with NHS. Moreover, we illustrated that skewed X‐chromosome inactivation and aberrant transcription were possible causes of significant phenotypic heterogeneity of NHS in carrier females. The novel variation in the *NHS* gene might highlight the understanding of the pathogenic mechanism of NHS and contribute to clinical Prenatal genetic diagnosis.

## AUTHOR CONTRIBUTIONS

Yazhou Huang analyzed the data and prepared the manuscript. Linya Ma, Zhaoxia Zhang, Shujuan Nie and Dan Peng collected the data. Jibo Zhang, Chao Wang, Xingxin Fang, Ting He and Anhui Liu rechecked the data. Dan Peng, Yuan Zhou, Linya Ma and Yingting Quan provided theoretical guidance. Dan Peng revised the manuscript and read and approved the final manuscript. All authors approved the final manuscript.

## CONFLICT OF INTEREST

The authors declare no conflict of interest.

## ETHICS STATEMENT

Written informed consent was obtained from the individual(s) for the publication of any potentially identifiable images or data included in this article.

## Data Availability

Data available on request from the authors.
